# High dietary inflammatory index is associated with an increased risk of overweight and obesity in adults: a meta-analysis of observational studies

**DOI:** 10.3389/fnut.2026.1881176

**Published:** 2026-06-30

**Authors:** Ruilian Gu, Meiping Qi, Huifeng Lu, Xinyi Miao, Yuting Wang, Xiaoxia Lin, Ran Wang, Zhenzhen Huang, Shufang Xia

**Affiliations:** 1 Department of Emergency Medicine, VIP Ward, Songjiang Hospital Affiliated to Shanghai Jiao Tong University School of Medicine, Shanghai, China; 2 Wuxi School of Medicine, Jiangnan University, Wuxi, Jiangsu, China

**Keywords:** dietary inflammatory index, meta-analysis, obesity, observational studies, overweight, systematic review

## Abstract

**Background and objective:**

Unhealthy dietary patterns are recognized risk factors for obesity by promoting chronic low-grade inflammation. This meta-analysis aimed to examine whether higher DII scores are associated with overweight/obesity in adults.

**Methods:**

PubMed, Embase, ScienceDirect, Cochrane Library, and Web of Science databases were searched up to April 16, 2025. Standardized mean differences (SMDs) and 95% confidence intervals (CIs) were pooled for the highest versus lowest DII.

**Results:**

A total of 22 studies were included, comprising 19 cross-sectional studies and three prospective cohort studies. Meta-analysis of the cross-sectional studies indicated a small but significant positive association between DII and BMI (Cohen’s d = 0.17; 95% CI: 0.09, 0.25), with significant heterogeneity (Q test, *p* < 0.001; *I*^2^ = 85.7%). After excluding one study identified by leave-one-out sensitivity analysis, the pooled effect size increased slightly and heterogeneity significantly decreased (Cohen’s d = 0.20; 95% CI: 0.17, 0.23; Q test, *p* = 0.032; *I*^2^ = 42.0%). Meta-regression analysis indicated that publication year and WHO regions partially explained heterogeneity (*p* = 0.016; *p* = 0.004), whereas subgroup analyses revealed only publication year as statistically significant (*p* = 0.040). Cohort studies generally suggested a positive association between DII and BMI (RR = 1.59, 95% CI: 0.72, 3.50; *β* = 0.41, 95% CI: 0.21, 0.61; HR = 1.32, 95% CI: 1.08, 1.60).

**Conclusion:**

Higher DII is associated with overweight/obesity. Further studies are needed to clarify the temporal relationship and underlying biological mechanisms linking dietary inflammatory potential with overweight/obesity.

**Systematic review registration:**

https://www.crd.york.ac.uk/PROSPERO/view/CRD420251049098, Identifier: CRD420251049098.

## 1 Introduction

Obesity, as a major public health problem, has imposed a significant global healthcare burden (1). It is also a critical risk factor for a variety of chronic diseases, such as cardiovascular diseases, diabetes, and cancer (2). According to data released by the World Health Organization (WHO) in 2022, approximately 2.5 billion adults aged 18 years and older were overweight, and more than 890 million adults were obese (3). Although there are inherent differences in body size and height among different populations (4), lifestyle factors such as dietary intake that influence energy balance are considered key determinants of the current obesity epidemic (5).

Multiple observational or interventional studies have shown that certain foods, nutrients, and dietary components are associated with body weight and obesity-related outcomes (6–8). The specific biological mechanisms by which diet affects obesity are not well defined and may involve oxidative stress (9), inflammation (10), gut microbiota (11), and epigenetic modifications (12). Among them, due to the chronic, low-grade inflammatory state of obesity, inflammation is thought to have a significant impact on energy balance and fat accumulation (13). It is well known that individual diets can influence systemic inflammation, which has been associated with obesity (14). Diet rich in fruits and vegetables with anti-inflammatory characteristics have been reported to be associated with a reduced risk of obesity (15, 16), while Western diets characterized by fast food, meat, fried foods, processed foods, and sugary beverages have been associated with pro-inflammatory states and obesity (17, 18). However, assessing the anti-inflammatory effects of individual nutrients or food components and their effects on weight does not fully reflect the interactions between dietary components or the complexity of the overall diet. Therefore, this may hinder its application in practical weight management.

The dietary inflammatory index (DII) was developed based on the association between 45 dietary components and food items with six inflammatory biomarkers through a comprehensive literature review of the studies published from 1950 to 2010 (19). The energy-adjusted dietary inflammatory index (E-DII), which adjusts the DII for total energy intake, has been used to improve comparability across individuals or populations with different energy intake levels (20). In fact, DII is the sum of the positive and negative scores attributed to each of the 45 dietary components and offers a quantitative method for evaluating the overall inflammatory potential of the diet in relation to health outcomes ranging from circulating inflammatory cytokines to chronic diseases, including central obesity (21), metabolic syndrome (22), cardiovascular diseases (23). Therefore, it could reflect all evidence from a wide variety of human populations using different study designs and dietary assessment methods. Previous studies have reported conflicting results. Cross-sectional studies showed that DII was positively associated with overweight/obesity (24, 25). A cohort study with 8 years of follow-up demonstrated that the DII was associated with weight gain and developing obesity in normal weight individuals (26). Another study showed that normal-weight participants had higher DII scores than obese participants (27), while other population-based studies did not detect any association between DII and body weight, body mass index (BMI), fat mass and abdominal obesity (28–31).

To develop nutrition policies, it is crucial to ascertain the association between DII and obesity. These findings may contribute to understanding the potential relationship between dietary inflammatory potential and obesity-related outcomes. To date, only a limited number of meta-analyses have examined the association between DII and obesity. However, substantial between-study heterogeneity has been reported, and potential sources of heterogeneity have not been fully explored. With the publication of additional observational studies, an updated synthesis using more comprehensive methodological approaches is warranted to provide a more complete assessment of the evidence and to further explore potential sources of heterogeneity. Therefore, this systematic review and meta-analysis updated the literature search to April 16, 2025, focused on adult cross-sectional and prospective cohort studies, excluded case–control studies, quantitatively synthesized cross-sectional evidence on the association between DII and BMI, and summarized prospective cohort evidence separately. Potential sources of heterogeneity were further explored through sensitivity analysis, subgroup analysis, and meta-regression. Information on dietary assessment tools, DII component parameters, and the use of DII or E-DII was also extracted to support a more detailed interpretation of methodological differences across studies. We hypothesized that higher DII scores, reflecting a more pro-inflammatory diet, are associated with overweight/obesity, and higher BMI in adults.

## 2 Methods

### 2.1 Protocol and registration

This systematic review and meta-analysis was performed adhering to the Preferred Reporting Items for Systematic Reviews and Meta-Analyses (PRISMA) statement (32) and following the guidance presented in *Cochrane Handbook* for Systematic Reviews of Interventions (33). The protocol has been registered with the International Prospective Register of Systematic Reviews (PROSPERO) under registration number CRD420251049098. The protocol is available online at https://www.crd.york.ac.uk/PROSPERO/view/CRD420251049098.

### 2.2 Search strategy

We systematically searched PubMed, Embase, ScienceDirect, Cochrane Library, and Web of Science from inception to April 16, 2025. Only studies published in English were included. A search strategy was developed using a combination of Medical Subject Headings (MeSH) terms and free-text words from the PubMed database, which included terms such as “dietary inflammatory index,” “dietary inflammatory potential,” “anti-inflammatory diet,” “pro-inflammatory diet,” “obesity,” “overweight,” “abdominal obesity,” “adult,” combined with appropriate Boolean operators (AND/OR). The search terms used for each electronic database are shown in Supplementary Table S1. Additionally, the reference lists of the retrieved articles were manually screened to identify any potentially eligible publications. Two researchers (R.G. and M.Q.) independently screened the titles and abstracts, and then assessed the full texts of relevant articles. To avoid overlapping data from the same cohort, only the study with the largest sample size and most recent publication year was included. Any disagreements were resolved through discussion between the two reviewers. If the disagreement remains unresolved, a third senior reviewer would participate and make the final decision.

### 2.3 Eligibility criteria

In this systematic review and meta-analysis, observational studies that assessed the association between DII and overweight/obesity were included with the PECOS (population, exposures, comparator, outcome, and study design) strategy (Table 1). The inclusion criteria were: (1) conducted in populations aged 18 years or older; (2) cohort and cross-sectional studies; (3) cross-sectional study reported odds ratio (OR) with corresponding 95% confidence interval (CI), or mean ± standard deviation (SD) of BMI along with sample sizes in the highest and lowest DII categories. Excluded studies comprised: (1) randomized controlled trial, case–control study, reviews, systematic reviews, meta-analyses, conference abstracts, book chapters, patents, case reports, and editorials/letters; (2) studies on children, adolescents, or special populations (e.g., pregnant women, cancer patients, etc.); (3) cross-sectional studies reporting *β* coefficients that could not be converted into standardized mean differences (SMDs); and (4) studies only reported other body composition indicators, including waist circumference, waist-to-hip ratio, body fat percentage, etc.

**Table 1:** PECOS criteria for inclusion and exclusion of studies.

Parameter	Inclusion criteria	Exclusion criteria
Population	Studies conducted in populations aged 18 years or older.	Studies involving children or adolescents; or special populations (e.g., pregnant women, cancer patients, etc.)
Exposure	Dietary Inflammatory Index (DII) or Dietary inflammatory potential or Anti-inflammatory diet or Pro-inflammatory diet.	Studies that did not use Dietary Inflammatory Index (DII) or Energy-adjusted Dietary Inflammatory Index (E-DII) to assess dietary inflammation; or did not clearly describe the method of DII calculation.
Comparison	Most pro-inflammatory vs. the most anti-inflammatory diets.	Studies that did not stratify by the DII score.
Outcome	Body mass index (BMI).	Other body composition indicators.
Study design	Cohort studies and cross-sectional studies.	Randomized controlled trial, case–control study, reviews, systematic reviews, meta-analyses, conference abstracts, book chapters, patents, case reports, and editorials/letters.

### 2.4 Data extraction

Data were collected according to a standard data extraction form independently by two reviewers. If a disagreement was reached, it would be resolved by a third reviewer. The extracted data from each eligible study were: first author’s name, year of publication, country in which the study was conducted, study design, sample size, age (mean ± SD or range), sex, follow-up duration (cohort studies), dietary assessment tools, number of DII components, DII categories, DII score comparison, effect size estimates, and potential confounder adjustments. For studies reporting categorical associations, we extracted odds ratios (OR) with corresponding 95% confidence intervals (CI). For studies reporting continuous outcomes, we extracted the mean ± standard deviation (SD) of BMI and the sample sizes in the highest and lowest DII categories in order to calculate standardized mean differences (SMD).

### 2.5 Quality assessment

The methodological quality of the included studies was independently assessed by two reviewers (R.G. and M.Q.) using the Quality Assessment Tool for Observational Cohort and Cross-Sectional Studies (34), disagreements between the researchers were discussed and resolved with a third researcher (Z.H.). This tool consists of 14 questions, each of which is answered with a positive response (yes). If the assessed item does not exist, cannot be determined, is not applicable, or is not reported, it is answered with a negative response (no). Studies were categorized into three levels of quality: Good quality: ≥ 75% of applicable items rated as “yes”; Fair quality: 50 to < 75% of applicable items rated as “yes”; Poor quality: < 50% of applicable items rated as “yes” (35). Studies rated as poor quality were excluded from this meta-analysis.

### 2.6 Statistical analysis

Due to the limited number of cohort studies included and different types of effect sizes and statistical models (e.g., *β* coefficients cannot be pooled with HRs or RRs), this meta-analysis was only conducted on cross-sectional studies. If the original studies reported the mean ± SD of BMI in the highest and lowest DII groups along with the corresponding sample sizes, the SMD was calculated using Cohen’s d method. When only the median, interquartile range (IQR), and sample size of each DII group were reported, the mean ± SD were estimated using the method proposed by Wan et al. (36). In brief, the mean was approximated by averaging the median and the quartiles, and the SD was estimated by dividing the IQR (i.e., Q3 − Q1) by 1.35, assuming a normal distribution of the data (36). If OR with 95% CI were reported, SMD were calculated using the method described by Chinn to get pooled effect size estimation (37). According to the empirical guidelines for effect sizes defined by Cohen, SMDs of 0.2, 0.5, and 0.8 represent small, medium, and large effects (38).

A random-effects model was constructed using the DerSimonian–Laird method to compute the pooled effect size and its 95% CI. Heterogeneity was assessed using the Q test and quantified by *I*^2^ test. As the Q test has low sensitivity when the number or sample size of included studies is small, a significance level of *p* < 0.10 (instead of 0.05) was used to improve detection sensitivity (33). A *p* value < 0.10 was considered indicative of significant heterogeneity, the degree of heterogeneity was further evaluated by *I*^2^ test, which ranges from 0 to 100% (39). Heterogeneity was considered as unimportant (*I*^2^ ≤ 25%), low (25% < *I*^2^ ≤ 50%), moderate (50% < *I*^2^ < 75%), or high (*I*^2^ ≥ 75%) (40). When *I*^2^ ≥ 50%, a random-effects model was used; otherwise, a fixed-effects model was used. To examine the robustness of the pooled result and to explore potential sources of heterogeneity, a leave-one-out sensitivity analysis was performed. To further explore sources of heterogeneity, subgroup analyses were conducted based on the following variables: WHO regions, population health status (healthy or unhealthy), dietary assessment tool (food frequency questionnaire, FFQ; 24-h dietary recall, 24-HDR; or FFQ plus 24-HDR), DII component parameters (≥30 or <30), DII grouping category (quartile, tertile or others), effect size category (OR or SMD), publication year (>2020 or ≤2020), and proportion of female participants (≤50% or >50%). The above variables were selected because they might affect the association between DII and overweight/obesity, based on *a priori* hypothesis and evidence from relevant literature. In addition, univariable meta-regression analyses were performed to explore the potential influence of the above variables on BMI. Publication bias was assessed by funnel plots and Egger’s test. All statistical analyses were performed using STATA version 18.0 (StataCorp LLC, College Station, TX, USA).

## 3 Results

### 3.1 Literature search

**Figure 1:**
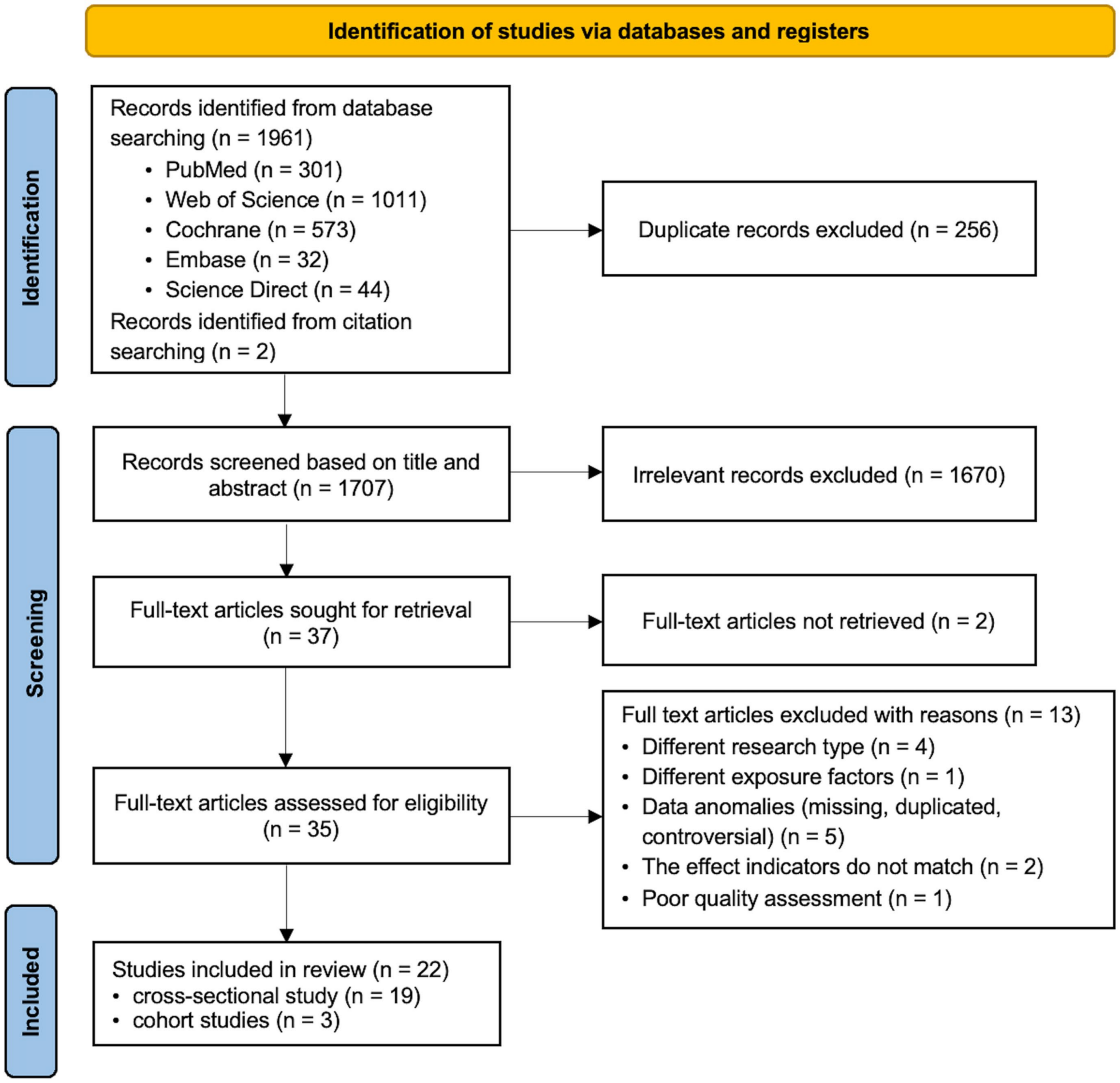
PRISMA 2020 flow diagram of study screening and selection process.

A total of 1,961 references were initially identified from databases. By screening the reference lists of relevant articles, an additional 2 references were retrieved. After excluding 256 duplicate references, the 1,707 publications remained for further evaluation. We reviewed the titles and abstracts of these articles and excluded the irrelevant references, 37 articles remained for full-text review based on the inclusion criteria, of which 22 eligible references were qualified for the final meta-analysis. The PRISMA flow diagram of study screening and selection process is shown in Figure 1.

A total of 22 studies were included, comprising 19 cross-sectional studies (25, 27, 29, 31, 41–55) and 3 prospective cohort studies (26, 56, 57). These studies involved 110,891 participants, including one study focusing exclusively on females (31), one on males (48), and 20 on both sexes. All participants were aged 18 years or older, with mean ages ranging from 20.41 to 67.15 years. The included studies were published between 2015 and 2025 and were conducted across multiple WHO regions, with the largest proportion from the Americas Region (*n* = 6) and the Eastern Mediterranean Region (*n* = 6), followed by the Western Pacific Region (*n* = 5), the European Region (*n* = 3), and the South-East Asia Region (*n* = 2). Assessments of dietary intake were mainly performed using FFQ (*n* = 12) and 24-HDR (*n* = 9), while one study used a combination of FFQ and 24-HDR. Among the included studies, three studies assessed individuals’ dietary inflammatory potential using the energy-adjusted dietary inflammatory index (E-DII), while the remaining studies applied the DII. The number of dietary components used to calculate the DII ranged from 23 to 45, with most studies including more than 28 components. In addition, DII scores was most categorized into quartiles (*n* = 11), followed by tertiles (*n* = 6), quintiles (*n* = 3), and dichotomization (*n* = 2). All cohort studies reported follow-up durations, with the mean follow-up duration ranging from 5 to 11.6 years. All studies that reported dichotomous outcomes adjusted for potential confounding variables, such as age, sex, and physical activity. The characteristics of the included studies are summarized in Table 2.

**Table 2:** Characteristics of the included studies (*n* = 22).

First author (year)	Country	Study design	Sample size	Age (mean ± SD, range)	Sex (male/female)	Duration of follow-ups (year)	No. of DII components	Dietary assessment tool	DII categories	DII comparison	Effect size (95% CI)	Adjusted variables
Darbandi et al. (2021) (41)	Iran	Cross-sectional study	8,520	47.24 ± 8.31	4275/4245	NA	31	FFQ	Quartile	Q4 vs. Q1 (−0.08 ± 1.05 vs. −4.04 ± 0.41)	Cohen’s d = 0.20 (0.14, 0.26)	/
Rezazadegan et al. (2024) (42)	Iran	Cross-sectional study	206	43.50 ± 8.82	61/146	NA	28	FFQ	Tertile	T3 vs. T1 (> − 1.59 vs. < −3.59)	Cohen’s d = 0.29 (−0.04, 0.63)	/
Karimbeiki et al. (2021) (27)	Iran	Cross-sectional study	300	36.00 ± 10.20	135/165	NA	30	FFQ	Tertile	T3 vs. T1	OR = 0.48 (0.26, 0.91)	1, 2, 10
Shi et al. (2023) (25)	USA	Cross-sectional study	10,723	40.80	5083/5640	NA	45	24-HDR	Quartile	Q4 vs. Q1 (2.95 ~ 5.50 vs. –4.63 ~ 0.06)	OR = 1.76 (1.50, 2.08)	1, 2, 3, 4, 5, 6, 7, 8
San et al. (2018) (31)	Myanmar	Cross-sectional study	244	25–60	0/244	NA	31	FFQ	Dichotomization (based on median)	Higher DII vs. Lower DII (>1.07 vs. <1.07)	OR = 1.4 (0.80, 2.30)	1, 6, 9, 20, 21
Li et al. (2024) (43)	USA	Cross-sectional study	5,152	53.25 ± 17.25	2563/2589	NA	28	24-HDR	Quartile	Q4 vs. Q1 (2.90 ~ 5.52 vs. –5.20 ~ −0.09)	OR = 1.56 (1.23, 1.98)	1, 2, 4, 5, 7, 22, 23
Ruiz-Canela et al. (2015) (44)	Spain	Cross-sectional study	7,236	67.15 ± 6.23	3091/4145	NA	45	FFQ	Quintile	Q5 vs. Q1 (male: 0.50 ~ 3.70 vs. –5.20 ~ −2.20; female: 0.70 ~ 3.70 vs. –4.90 ~ −2.00)	Cohen’s d = 0.17 (0.10, 0.24)	/
Mazidi et al. (2018) (45)	USA	Cross-sectional study	17,689	45.80	8544/9145	NA	39	24-HDR	Quartile	Q4 vs. Q1 (1.62 ~ 4.24 vs. –5.66 ~ −1.04)	OR = 1.40 (1.22, 1.60)	1, 2, 3, 5, 6, 7, 11
Muhammad et al. (2019) (29)	Indonesia	Cross-sectional study	503	41.60 ± 10.20	252/251	NA	30	FFQ	Tertile	T3 vs. T1 (>5.10 vs. < −1.00)	Cohen’s d = 0.04 (−0.18, 0.25)	/
Correa-Rodríguez et al. (2018) (46)	Spain	Cross-sectional study	599	20.41 ± 2.72	185/414	NA	25	24-HDR	Quartile	Q4 vs. Q1 (>1.21 ~ 2.52 vs. –4.02 ~ ≤ − 0.48)	Cohen’s d = −0.04 (−0.27, 0.19)	/
Togug et al. (2025) (47)	Turkey	Cross-sectional study	124	42.20 ± 11.00	49/75	NA	32	24-HDR	Quartile	Q4 vs. Q1 (3.01 ± 0.44 vs. −0.15 ± 0.60)	Cohen’s d = 0.46 (−0.05, 0.96)	/
Corrêa et al. (2022) (48)	Brazil	Cross-sectional study	59	26.20 ± 4.20	59/0	NA	28	24-HDR	Tertile	T3 vs. T1 (1.46 ~ 3.10 vs. –3.48 ~ 0.25)	Cohen’s d = 0.67 (0.02, 1.31)	/
Zhang et al. (2024) (49)	China	Cross-sectional study	1,193	45.18 ± 15.01	403/790	NA	45	FFQ + 24-HDR	Quartile	Q4 vs. Q1 (>2.51 vs. < −2.03)	OR = 1.06 (0.76, 1.48)	1, 2, 26
Zhao et al. (2023) (51)	USA	Cross-sectional study	3,843	≥45	1887/1956	NA	27	24-HDR	Quartile	Q4 vs. Q1 (2.05 ~ 4.66 vs. –5.20 ~ −1.00)	Cohen’s d = 0.25 (0.16, 0.34)	/
Rahimlou et al. (2024) (52)	Iran	Cross-sectional study	2045	53.15 ± 8.93	737/1308	NA	45	FFQ	Tertile	T3 vs. T1 (1.13 ± 0.87 vs. –3.61 ± 0.87)	Cohen’s d = 0.30 (0.19, 0.40)	/
Mokhtary et al. (2020) (53)	Iran	Cross-sectional study	726	50.07 ± 6.42	280/446	NA	15	FFQ	Tertile	T3 vs. T1	Cohen’s d = 0.07 (−0.11, 0.24)	/
Camargo-Ramos et al. (2017) (50)	Colombia	Cross-sectional study	90	39.70 ± 6.90	Both (numbers unspecified)	NA	28	24-HDR	Dichotomization	Pro-inflammatory diet vs. Anti-inflammatory diet (−0.13 ~ 3.64 vs. –3.71 ~ −0.37)	Cohen’s d = 0.37 (−0.22, 0.96)	/
Kim et al. (2018) (54)	South Korea	Cross-sectional study	9,291	41.30 ± 0.20	3682/5609	NA	23	24-HDR	Quartile	Q4 vs. Q1 (male: ≥1.89 vs. < −0.16; female: ≥1.28 vs. < −0.85)	Cohen’s d = −0.13 (−0.19, −0.07)	
Shu et al. (2022) (55)	China	Cross-sectional study	6,730	62.87 ± 7.99	2774/3956	NA	26	FFQ	Quartile	Q4 vs. Q1 (1.73 ~ 3.90 vs. –5.84 ~ −1.53)	Cohen’s d = 0.15 (0.08, 0.21)	
Wang et al. (2021) (56)	Australia	Cohort study	787	≥18	361/426	5	29	FFQ	Quintile	Q5 vs. Q1	RR = 1.59 (0.72, 3.50)	1, 2, 5, 6, 7, 8, 9, 24
Hodge et al. (2021) (57)	Australia	Cohort study	27,804	54.30 ± 8.60	11,030/16774	11.6	29	FFQ	Quintile	Q5 vs. Q1 (1.30 ~ 2.60 vs. –3.30 ~ −2.70)	β = 0.41 (0.21, 0.61)	1, 2, 7, 8, 9, 10, 11, 24, 25
Ramallal et al. (2017) (26)	Spain	Cohort study	7,027	37.40 ± 10.50	2459/4568	8.1	45	FFQ	Quartile	Q4 vs. Q1 (−0.59 ~ 4.00 vs. –5.10 ~ −2.50)	HR = 1.32 (1.08, 1.60)	1, 2, 7, 8, 9, 11, 12, 13, 14, 15, 16, 17, 18, 19, 27, 28, 29

Adjusted for: 1 = age; 2 = sex; 3 = race / ethnicity; 4 = family poverty income ratio; 5 = education; 6 = marital status; 7 = smoking status; 8 = alcohol use; 9 = physical activity; 10 = waist circumference /waist-to-hip ratio; 11 = energy intake; 12 = baseline BMI; 13 = hours of TV watching; 14 = sitting time; 15 = snacking between meals; 16 = special diet at baseline; 17 = family history of obesity (parents); 18 = siesta; 19 = depression; 20 = contraception; 21 = parity; 22 = hypertension; 23 = diabetes; 24 = socioeconomic status; 25 = country of birth; 26 = occupation; 27 = analgesics use; 28 = antidepressant/anxiolytic therapy; 29 = antihypertensive therapy.

OR, odds ratio; SMD, standardized mean difference (Cohen’s d); CIs, confidence intervals; FFQ, food frequency questionnaire; 24-HDR, 24-h dietary recall; NA, not applicable; Mean ± SD, mean ± standard deviation.

### 3.2 Quality assessment

According to the Quality Assessment Tool for Observational Cohort and Cross-Sectional Studies, one study was rated as poor quality and excluded from this study (58); another was rated as high quality (55), and the remaining 21 studies were rated as fair quality (25–27, 29, 31, 41–55, 57). The detailed results of quality assessment are shown in Supplementary Table S2.

### 3.3 Association between DII and the risk of overweight/obesity

The three prospective cohort studies were not included in the meta-analysis due to differences in effect size measurement methods. As shown in Table 2, all three cohort studies showed associations in the same direction between higher DII scores and overweight/obesity outcomes (26, 56, 57). A cohort study conducted in Australia followed 11,030 men and 16,774 women for an average of 11.6 years, showing that each 1-unit increase in the DII was associated with a 0.41-unit increase in BMI (*β* = 0.41; 95% CI: 0.21, 0.61; *p* < 0.001) (57). Another cohort study involving 7,027 Spanish university graduates with baseline BMI < 25 found that participants in the highest DII group had a 32% higher risk of developing obesity compared to those in the lowest DII group (HR = 1.32; 95% CI: 1.08, 1.60; *p* = 0.011) (26). In addition, a 5-year follow-up study among 787 healthy adults found that individuals in the highest DII group had a 1.59-fold increased risk of developing obesity compared to those in the DII lowest group, though this association did not reach statistical significance (RR = 1.59; 95% CI: 0.72, 3.50; *p* = 0.06) (56). Taken together, the results of these prospective studies indicate a generally consistent trend in the association between pro-inflammatory diets and obesity risk.

**Figure 2:**
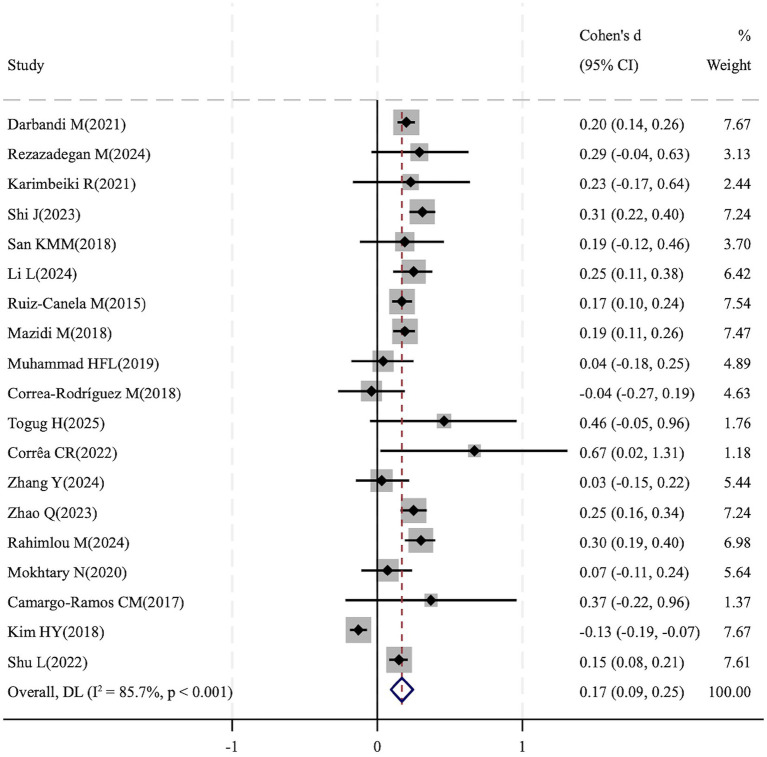
Forest plot of the association between the dietary inflammatory index and BMI (highest vs. lowest DII groups). Weights are from random-effects model.

The association between DII and overweight/obesity is shown in Figure 2. The pooled analysis on 19 cross-sectional studies revealed a small but significant positive association between DII scores and BMI (Cohen’s d = 0.17; 95% CI: 0.09, 0.25), with high heterogeneity (Q test, *p* < 0.001; *I*^2^ = 85.7%).

**Figure 3:**
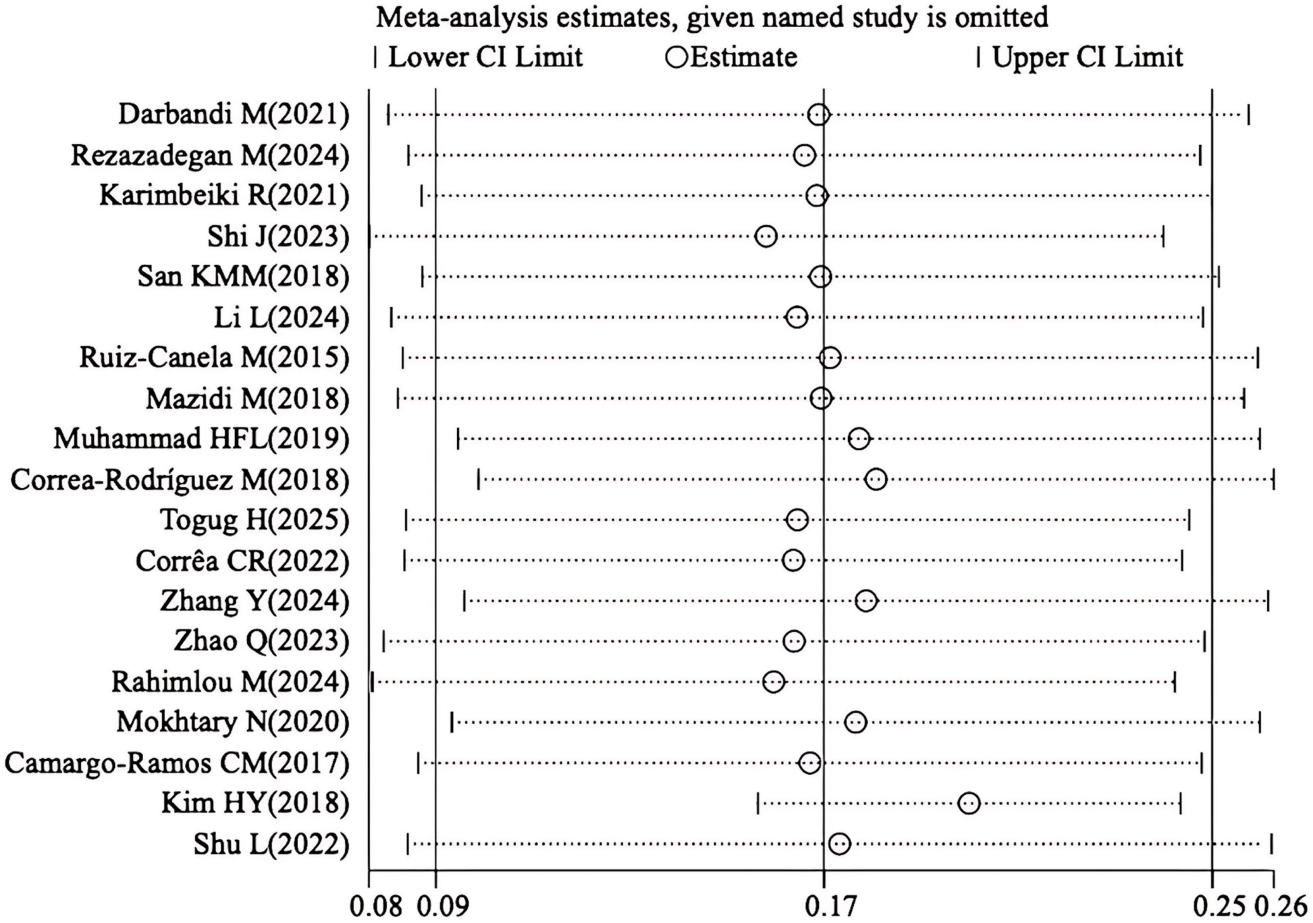
Sensitivity analysis plot assessing the robustness of the association between the dietary inflammatory index and BMI (highest vs. lowest DII groups).

**Figure 4:**
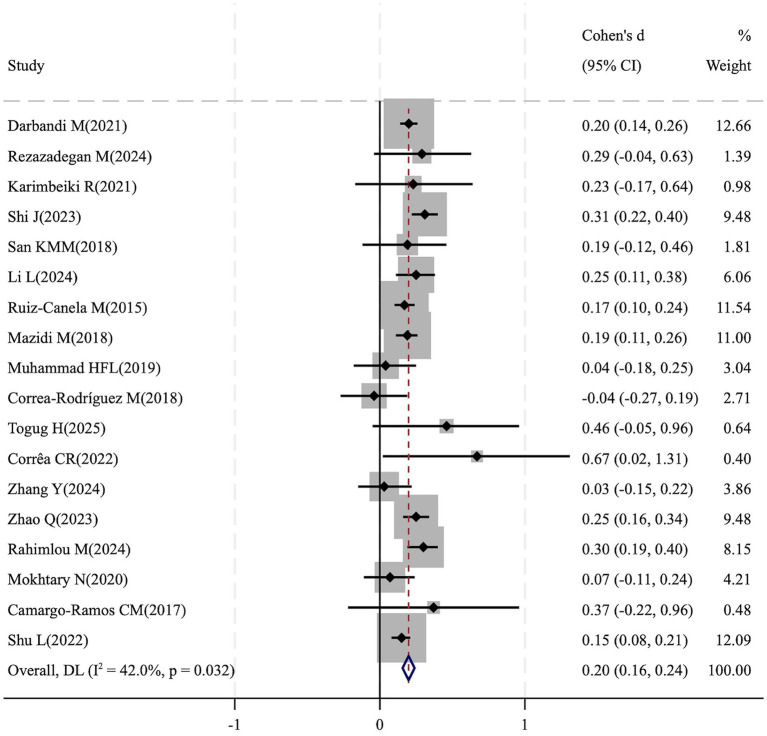
Forest plot of the association between the dietary inflammatory index and BMI after excluding one study identified as a potential source of high heterogeneity (highest vs. lowest DII groups). Weights are from random-effects model.

Sensitivity analysis indicated that the study by Kim et al. (54) had a significant impact on the overall heterogeneity among the studies (Figure 3). After excluding this study, the pooled effect size slightly increased (Figure 4; Cohen’s d = 0.20; 95% CI: 0.17, 0.23) and the heterogeneity was low (*I*^2^ = 42.0%; Q test, *p* = 0.032). This discrepancy may arise because that study reported a lower mean BMI in the highest DII group compared with the lowest DII group, whereas the other studies showed the opposite finding.

To assess the effects of grouping factors and identify sources of heterogeneity, subgroup analyses were conducted based on the WHO regions, population health status, dietary assessment tool, DII component parameters, DII grouping category, effect size category, publication year, and proportion of female participants. As shown in Supplementary Table S3, subgroup analysis stratified by publication year showed statistically significant differences between groups (*p* = 0.040). Studies published after 2020 demonstrated a pooled effect size of Cohen’s d = 0.23 (Supplementary Figure S1, 95% CI: 0.18, 0.28; Q test, *p* = 0.047), with low heterogeneity (*I*^2^ = 46.0%); whereas those published in 2020 or earlier yielded a pooled effect size of Cohen’s d = 0.08 (95% CI, −0.05, 0.21, Q test, *p* < 0.001), with high heterogeneity (*I*^2^ = 88.7%). Differences by WHO regions approached statistical significance (*p* = 0.08). Analysis based on other variables indicated that subgroup factors did not have a significant effect on the outcomes (all *p* > 0.05) and did not reveal sources of heterogeneity.

Univariable meta-regression analyses were also performed to explore the sources of heterogeneity (Supplementary Table S4). The results showed that year of publication was statistically significant (*β* = 0.160; 95% CI: 0.03, 0.29; *p* = 0.016), explaining 34.39% of the observed heterogeneity (adjusted *R*^2^ = 34.39%). WHO region was also statistically significant (β = −0.060; 95% CI: −0.10, −0.02; *p* = 0.004), accounting for 48.94% of the between-study heterogeneity (adjusted *R*^2^ = 48.94%). The regression coefficients for the remaining covariates—including effect size type, population health status, dietary assessment tool, female proportion, DII component parameters, and sample size—were not statistically significant (all *p* > 0.05), with adjusted *R*^2^ values close to or below zero.

### 3.4 Publication bias

Slight asymmetry was observed in the funnel plot, suggesting potential heterogeneity or publication bias (Supplementary Figure S2). However, Egger’s test did not indicate significant small-study effects or publication bias (intercept = 0.963; 95% CI: −1.36, 3.28; *p* = 0.39).

## 4 Discussion

This systematic review and meta-analysis was conducted to investigate the association between DII and overweight/obesity in adults. Our findings were generally consistent with the hypothesis that higher DII scores are associated with overweight/obesity and higher BMI in adults. The findings of 19 cross-sectional studies showed that a higher dietary pro-inflammatory potential indicated by a higher DII score was positively associated with increased BMI and a greater risk of overweight/obesity. It is worth noting that the systematic review of the included prospective cohort studies also demonstrated a consistent positive association between DII scores and the risk of overweight/obesity, thereby strengthening the credibility of this association from a longitudinal perspective.

Although inflammation is commonly considered as a consequence of obesity, previous studies have reported that inflammatory processes are associated with obesity development and weight gain (49). Abnormal circulating inflammatory biomarkers, such as tumor necrosis factor-α (TNF-α), interleukin-6 (IL-6), or C-reactive protein (CRP) have been reported in overweight and obese adults (59). Cohort studies found that elevated CRP levels were correlated with an increased risk of future weight gain (60, 61). Diet has been proved to play an important role in the progression of inflammation, with certain foods and nutrients being capable of eliciting immunomodulatory effects, and thus may affect obesity. A pro-inflammatory diet could upregulate the expression of IL-6, CRP and TNF-α (19, 62), and may contribute to adipocyte dysfunction, fat accumulation, and an increased risk of obesity (63). In contrast, anti-inflammatory dietary components (such as *ω*-3 fatty acids) inhibited the release of CRP, thereby preventing the onset and progression of obesity (64). A cross-sectional study involving 794 overweight and obese participants from the PREDIMED trial revealed that intake of anti-inflammatory nutrients or components (such as polyunsaturated fats, ω-3 and ω-6 fatty acids, vitamin E and D) was higher in subjects with a BMI ≤ 26.9 kg/m^2^, while intake of red meat, a source of pro-inflammatory saturated fats, was higher in obese participants (65). Additionally, animal experiment also showed that anti-inflammatory components in the diet, such as polyphenols, polysaccharides or caffeine, could reduce IL-6 and TNF-α levels in obese rats (66). Therefore, dietary patterns characterized by lower intake of pro-inflammatory foods and higher intake of anti-inflammatory foods may be relevant to obesity. However, future prospective cohort studies and well-designed intervention trials are needed to clarify the nature and strength of these associations.

Several observational studies have evaluated the association between the DII and indices for assessing overweight/obesity, including BMI, waist circumference and fat mass (24). In the Multiethnic Cohort-Adiposity Phenotype Study, there were positive total effects between E-DII and total fat mass, visceral adipose tissue in participants aged 60–77 years (67). A hospital-based case control study in Malaysia showed that as the E-DII grew, subjects’ mean values of BMI, waist circumference, waist-to-hip ratio, and body fat percentage significantly increased, and subjects in the highest quartile were more likely to be obese than those in the lowest quartile, whereas non-obese subjects were more likely to be in the lowest E-DII quartile (68). As a substitute for visceral adipose tissue and can be measured easily, BMI is commonly used in epidemiological studies (69). Results from the NHANES study 2007–2016 showed that individuals with higher DII scores were more likely to have higher BMI (70). From the same database 2007–2018, Zhang et al. (71) also found that DII was positively associated with BMI and waist circumference in 8,180 subjects. In middle-aged and elderly individuals in the United States, DII was also positively correlated with BMI (51). The North West Adelaide Health Study reported that the lower quintile of DII was correlated with a lower risk of obesity (56). A meta-analysis of cross-sectional studies showed that the pooled OR for the risk of abdominal obesity was significant in the highest DII group compared to the lowest group (22). However, research findings on the association between DII and overweight/obesity are not always consistent. Two cross-sectional studies from South Korea and Iran showed an inverse or no significant association between DII and obesity (54, 58). Data from the Korea National Health and Nutrition Examination Survey reported that the top DII quartile was not significantly associated with central obesity (54). The study among Iranian adults indicated that DII scores were significantly negatively associated with general obesity, but not significantly correlated with abdominal obesity (58). Our meta-analysis of 19 included cross-sectional studies showed a small but significant positive association between DII and BMI in adults, with high heterogeneity. After excluding the South Korean study by a leave-one-out sensitivity analysis, the aforementioned association remained significant, and heterogeneity decreased from high to low levels. We speculate that this might be related to regional differences, as variations in traditional dietary patterns, food composition, and nutrient availability across populations may influence the calculation of the DII and contribute to the observed heterogeneity among studies. Overall, the association remained robust after exclusion of this study; however, the substantial reduction in heterogeneity suggests that the pooled estimate was influenced by this individual study. Therefore, the findings should be interpreted with caution.

Prospective longitudinal studies further suggested a longitudinal association between DII and BMI. The SUN cohort study showed that 7,027 university graduates with highest quartile DII scores experienced a higher average yearly weight gain and a higher risk of developing new-onset overweight or obesity compared to those in the lowest quartile after a median follow-up of 8.1 years (26). An individual participant data pooled analysis of seven European cohorts found that maternal diets with higher E-DII throughout pregnancy were associated with a higher risk of late-childhood overweight and obesity (72). In the present study, only three cohort studies met the inclusion criteria, and the lack of information limited further meta-analysis. However, all these three studies showed that a high DII score was associated with increased BMI, further supporting a possible association between DII and obesity. But the large prospective Supplementation en Vitamines et Mineraux AntioXydants (SUVIMAX) cohort study in France failed to observe the significant association between DII and waist circumference in 3,726 subjects after an average follow-up of 12.4 years (73). These inconsistent findings from the cross-sectional and prospective longitudinal studies might be partly due to differences in study populations, obesity criteria, age, sex, socioeconomic status, dietary assessment tools, the number and types of food components and nutrients included in the DII calculation, and whether dietary supplements were recorded. Compared with the FFQ method, the 24-HDR method cannot reflect long-term dietary intake, especially some studies only collected data from a single 24-h dietary intake, which inevitably affect the DII calculation to a certain extent. The DII calculation method includes 45 DII food parameters, and the more food parameters included, the more accurately the dietary inflammatory potential can be reflected. Most of the literature included in the present study used 26–32 food parameters, but some studies used up to 45 parameters, resulting in a lack of consistency in the DII among different studies. Some anthropometric measurements used in some studies, such as weight, height, and waist circumference, were obtained through self-reporting rather than direct objective measurement, which may have introduced measurement bias (58). Additionally, dietary surveys on Western populations have found that recording the weight of subjects could introduce subjective bias into subsequent dietary survey data (74, 75). All these issues might explain the inconsistent association between DII and obesity.

In addition to observational studies, clinical intervention studies have also revealed the beneficial effects of an anti-inflammatory diet on weight loss. A Croatian intervention study found that 81 obese patients who followed a 24-week low-energy anti-inflammatory diet plan experienced significant reductions in weight, waist circumference, and visceral fat (76). Another study targeting overweight or obese women with polycystic ovary syndrome showed that patients who followed an anti-inflammatory diet combined with exercise intervention lost approximately 7% of their body weight (77). These findings suggest that anti-inflammatory dietary interventions may have potential value in weight management among individuals with overweight or obesity. Further clinical studies are warranted to better understand these findings, clarify the specific contribution of dietary intervention, and evaluate long-term effectiveness.

Subgroup analyses indicated that publication year was an important source of heterogeneity among studies. Studies published after 2020 reported higher pooled effect sizes and lower heterogeneity. Meta-regression further supported this finding, showing that for each additional year, the effect size increased by an average of 0.160, with this variable accounting for approximately 34.39% of the observed heterogeneity. This trend may reflect improvements in the DII scoring methodology (20), enhancements in the quality of nutritional epidemiology research (78) and evolving dietary patterns over time (79). Hébert et al. (20) summarized lessons learned and methodological refinements to the DII in 2019, highlighting algorithmic improvements such as literature-derived inflammatory effect scores and weights for up to 45 dietary components, standardization against global intake distributions, and treatment of total energy and nutrient density. Meta-regression by WHO regions showed that the regional variable was significantly associated with the effect size, suggesting that regional differences could largely account for the overall heterogeneity, while subgroup analysis based on WHO regions did not reveal statistically significant differences. Although DII has been standardized based on dietary intake data from multiple countries to facilitate comparisons across populations, previous studies have shown that differences in dietary patterns across geographic regions can still influence the distribution of DII scores and their associations with health outcomes. For example, the strength of the association between DII and metabolic syndrome varies among different WHO regions (22), and even within the same country, DII scores may differ across regions (80). Therefore, caution is warranted when interpreting DII in cross-regional meta-analyses. These findings partially support our earlier speculation. The study by Kim et al. (54) from South Korea was published earlier than most others, and subsequent improvements in DII algorithms and dietary assessment methods may explain the stronger and more consistent associations observed in recent researches. In addition, regional differences—such as dietary patterns, completeness of food databases, and population characteristics (including genetic background, lifestyle, and baseline BMI)—may also further affect the estimation of DII and contribute to the observed heterogeneity. It should be noted that although DII has broad applicability, its calculation is complex, and differences in regional dietary patterns may affect the selection of included components. Therefore, adopting updated and more comprehensive DII algorithms may enhance its accuracy and cross-population comparability.

### 4.1 Limitations

Some limitations should be noted. First, only English-language publications were included, which may have introduced language bias. Second, although we attempted to account for potential confounders, residual confounding could not be excluded. Because the quantitative meta-analysis was based on cross-sectional studies, causal relationships cannot be inferred, and temporality between DII and overweight/obesity could not be established. In addition, reverse causality cannot be excluded, as obesity status may influence dietary behaviors or dietary reporting. Third, dietary assessment tools, both dietary recalls and FFQs are potential sources of recall bias. The number of items in FFQ ranges from 63 to 168, and difference in the number of items in FFQ, as well as whether the questionnaires include items for local specialty foods, might be sources of heterogeneity (81, 82). In addition, differences in the global distribution of food resources, dietary patterns, and food cultures may lead to variation in the number of food components or nutrients included in DII calculations. Together with differences in energy adjustment and the use of DII versus E-DII, these methodological variations may have influenced the comparability of results across studies. Finally, BMI could not discriminate body fat and muscle mass. In the future, longitudinal studies or well-designed anti-inflammatory diet intervention program, with more objective obesity indicators, such as body fat percentage, standardized methods of DII calculation are needed to further clarify the association between DII and overweight/obesity.

### 4.2 Implications for research and practice

Our findings confirm a positive association between DII and the risk of overweight and obesity in adults. The heterogeneity observed across WHO regions suggests that regional dietary patterns, differences in food composition databases, sociodemographic characteristics, and methodological factors may modify this association. To strengthen causal inference, future randomized controlled trials and prospective cohort studies should at minimum: (1) implement longer-term interventions and follow-up to evaluate whether long-term changes in DII are associated with subsequent changes in body weight and adiposity; and (2) enroll geographically and ethnically diverse populations with pre-specified subgroup analyses by age and sex to assess generalizability and effect modification.

Potential mechanistic research is needed to clarify whether and how DII may be related to adiposity via systemic inflammation, alterations in the gut microbiota, insulin resistance, and related metabolic pathways. If future evidence supports these associations, culturally adapted anti-inflammatory dietary guidelines and community-based nutrition education programs may improve feasibility and uptake across settings. Finally, integrating DII with other lifestyle factors (e.g., physical activity and sleep) into a weighted composite score could help explore combined lifestyle patterns associated with overweight/obesity.

## 5 Conclusion

In conclusion, this systematic review and meta-analysis suggested that DII was positively associated with overweight/obesity in adults. In the future, more clinical trials and population-based prospective cohort studies are needed to further clarify the temporal relationship and potential biological pathways linking DII with overweight/obesity, and to identify anti-inflammatory dietary components that may be relevant to overweight/obesity.

## Data Availability

The original contributions presented in the study are included in the article/Supplementary material, further inquiries can be directed to the corresponding authors.

## References

[1] Afshin A Forouzanfar MH Reitsma MB Sur P Estep K Lee A . Health effects of overweight and obesity in 195 countries over 25 years. N Engl J Med. (2017) 377:13–27. doi: 10.1056/NEJMoa1614362, 28604169 PMC5477817

[2] GBD 2021 Risk Factors Collaborators. Global burden and strength of evidence for 88 risk factors in 204 countries and 811 subnational locations, 1990-2021: a systematic analysis for the global burden of disease study 2021. Lancet. (2024) 403:2162–203. doi: 10.1016/s0140-6736(24)00933-4, 38762324 PMC11120204

[3] World Health Organization. Obesity and overweight. Available online at: https://www.who.int/news-room/fact-sheets/detail/obesity-and-overweight (Accessed 21 June 2025).

[4] Qasim A Turcotte M de Souza RJ Samaan MC Champredon D Dushoff J . On the origin of obesity: identifying the biological, environmental and cultural drivers of genetic risk among human populations. Obes Rev. (2018) 19:121–49. doi: 10.1111/obr.12625, 29144594

[5] Adolph TE Tilg H. Western diets and chronic diseases. Nat Med. (2024) 30:2133–47. doi: 10.1038/s41591-024-03165-6, 39085420

[6] Nguyen M Jarvis SE Tinajero MG Yu J Chiavaroli L Mejia SB . Sugar-sweetened beverage consumption and weight gain in children and adults: a systematic review and meta-analysis of prospective cohort studies and randomized controlled trials. Am J Clin Nutr. (2023) 117:160–74. doi: 10.1016/j.ajcnut.2022.11.008, 36789935

[7] Kim JY. Optimal diet strategies for weight loss and weight loss maintenance. J Obes Metab Syndr. (2021) 30:20–31. doi: 10.7570/jomes20065, 33107442 PMC8017325

[8] Kristoffersen E Hjort SL Thomassen LM Arjmand EJ Perillo M Balakrishna R . Umbrella review of systematic reviews and meta-analyses on the consumption of different food groups and the risk of overweight and obesity. Nutrients. (2025) 17:662. doi: 10.3390/nu17040662, 40004990 PMC11857968

[9] Almoraie NM Shatwan IM. The potential effects of dietary antioxidants in obesity: a comprehensive review of the literature. Healthcare (Basel). (2024) 12:416. doi: 10.3390/healthcare12040416, 38391792 PMC10887832

[10] Mello RND Gois BPD Kravchychyn ACP Dâmaso AR Horst MA Lima GC . Dietary inflammatory index and its relation to the pathophysiological aspects of obesity: a narrative review. Arch Endocrinol Metab. (2023) 67:e000631. doi: 10.20945/2359-3997000000631, 37364142 PMC10661000

[11] Corbin KD Igudesman D Smith SR Zengler K Krajmalnik-Brown R. Targeting the gut microbiota’s role in host energy absorption with precision nutrition interventions for the prevention and treatment of obesity. Nutr Rev. (2025) 83:1928–43. doi: 10.1093/nutrit/nuaf046, 40233201 PMC12422011

[12] Gao W Liu J-L Lu X Yang Q. Epigenetic regulation of energy metabolism in obesity. J Mol Cell Biol. (2021) 13:480–99. doi: 10.1093/jmcb/mjab043, 34289049 PMC8530523

[13] Khanna D Khanna S Khanna P Kahar P Patel BM. Obesity: a chronic low-grade inflammation and its markers. Cureus. (2022) 14:e22711. doi: 10.7759/cureus.22711, 35386146 PMC8967417

[14] Yu X Pu H Voss M. Overview of anti-inflammatory diets and their promising effects on non-communicable diseases. Br J Nutr. (2024) 132:898–918. doi: 10.1017/s0007114524001405, 39411832 PMC11576095

[15] López-Gil JF García-Hermoso A Sotos-Prieto M Cavero-Redondo I Martínez-Vizcaíno V Kales SN. Mediterranean diet-based interventions to improve anthropometric and obesity indicators in children and adolescents: a systematic review with meta-analysis of randomized controlled trials. Adv Nutr. (2023) 14:858–69. doi: 10.1016/j.advnut.2023.04.011, 37127186 PMC10334150

[16] Remde A DeTurk SN Almardini A Steiner L Wojda T. Plant-predominant eating patterns—how effective are they for treating obesity and related cardiometabolic health outcomes? – a systematic review. Nutr Rev. (2021) 80:1094–104. doi: 10.1093/nutrit/nuab060, 34498070

[17] Clemente-Suárez VJ Beltrán-Velasco AI Redondo-Flórez L Martín-Rodríguez A Tornero-Aguilera JF. Global impacts of western diet and its effects on metabolism and health: a narrative review. Nutrients. (2023) 15:2749. doi: 10.3390/nu15122749, 37375654 PMC10302286

[18] Eng JY Moy FM Bulgiba A Rampal S. Dose-response relationship between western diet and being overweight among teachers in Malaysia. Nutrients. (2020) 12:3092. doi: 10.3390/nu12103092, 33050612 PMC7601593

[19] Shivappa N Steck SE Hurley TG Hussey JR Hébert JR. Designing and developing a literature-derived, population-based dietary inflammatory index. Public Health Nutr. (2014) 17:1689–96. doi: 10.1017/s1368980013002115, 23941862 PMC3925198

[20] Hébert JRSN Wirth MD Hussey JR Hurley TG. Perspective: the dietary inflammatory index (DII)-lessons learned, improvements made, and future directions. Adv Nutr. (2019) 10:185–95. doi: 10.1093/advances/nmy071, 30615051 PMC6416047

[21] Sokol A Wirth MD Manczuk M Shivappa N Zatonska K Hurley TG . Association between the dietary inflammatory index, waist-to-hip ratio and metabolic syndrome. Nutr Res. (2016) 36:1298–303. doi: 10.1016/j.nutres.2016.04.004, 27865615 PMC5119948

[22] Bakhshimoghaddam F Chaharlang R Mansoori A Dehghanseresht N. Dietary inflammatory index and its association with risk of metabolic syndrome and its components: a systematic review and meta-analysis of observational studies. J Health Popul Nutr. (2024) 43:87. doi: 10.1186/s41043-024-00580-w, 38898498 PMC11188268

[23] Vissers LE Waller MA van der Schouw YT Hebert JR Shivappa N Schoenaker DA . The relationship between the dietary inflammatory index and risk of total cardiovascular disease, ischemic heart disease and cerebrovascular disease: findings from an Australian population-based prospective cohort study of women. Atherosclerosis. (2016) 253:164–70. doi: 10.1016/j.atherosclerosis.2016.07.929 27498398

[24] Gholamalizadeh M Ahmadzadeh M BourBour F Vahid F Ajami M Majidi N . Associations between the dietary inflammatory index with obesity and body fat in male adolescents. BMC Endocr Disord. (2022) 22:115. doi: 10.1186/s12902-022-01001-x, 35501761 PMC9059349

[25] Shi J Liang Z Zhang X Ren S Cheng Y Liu Y . Association of physical activity and dietary inflammatory index with overweight/obesity in US adults: NHANES 2007-2018. Environ Health Prev Med. (2023) 28:40. doi: 10.1265/ehpm.23-00016, 37380500 PMC10331001

[26] Ramallal R Toledo E Martínez JA Shivappa N Hébert JR Martínez-González MA . Inflammatory potential of diet, weight gain, and incidence of overweight/obesity: the SUN cohort. Obesity (Silver Spring). (2017) 25:997–1005. doi: 10.1002/oby.21833, 28544794

[27] Karimbeiki R Alipoor E Yaseri M Shivappa N Hebert JR Hosseinzadeh-Attar MJ. Association between the dietary inflammatory index and obesity in otherwise healthy adults: role of age and sex. Int J Clin Pract. (2021) 75:e14567. doi: 10.1111/ijcp.14567, 34165878

[28] Jung S Lee Y Kim K Park S. Association of the dietary inflammatory index with sarcopenic obesity and frailty in older adults. BMC Geriatr. (2024) 24:654. doi: 10.1186/s12877-024-05239-z, 39097690 PMC11297761

[29] Muhammad HFL van Baak MA Mariman EC Sulistyoningrum DC Huriyati E Lee YY . Dietary inflammatory index score and its association with body weight, blood pressure, lipid profile, and leptin in indonesian adults. Nutrients. (2019) 11:148. doi: 10.3390/nu11010148, 30641979 PMC6356884

[30] Ghorabi S Esteghamati A Azam K Daneshzad E Sadeghi O Salari-Moghaddam A . Association between dietary inflammatory index and components of metabolic syndrome. J Cardiovasc Thorac Res. (2020) 12:27–34. doi: 10.34172/jcvtr.2020.05, 32211135 PMC7080330

[31] San KMM Fahmida U Wijaksono F Lin H Zaw KK Htet MK. Chronic low grade inflammation measured by dietary inflammatory index and its association with obesity among school teachers in Yangon, Myanmar. Asia Pac J Clin Nutr. (2018) 27:92–8. doi: 10.6133/apjcn.042017.06, 29222885

[32] Page MJ McKenzie JE Bossuyt PM Boutron I Hoffmann TC Mulrow CD . The PRISMA 2020 statement: an updated guideline for reporting systematic reviews. BMJ. (2021) 372:n71. doi: 10.1136/bmj.n71, 33782057 PMC8005924

[33] Higgins JPT Thomas J Chandler J Cumpston M Li T Page MJ . Cochrane Handbook for systematic Reviews of Interventions Version 6.5 (Updated August 2024). Cochrane; (2024). Available online at: https://training.cochrane.org/handbook (Accessed on August 9, 2025).

[34] National Institutes of Health DoHaHS. Quality assessment tool for observational cohort and cross-sectional studies. Available online at: https://www.nhlbi.nih.gov/health-topics/study-quality-assessment-tools (Accessed August 9, 2025).

[35] Pizarro-Pennarolli C Sánchez-Rojas C Torres-Castro R Vera-Uribe R Sanchez-Ramirez DC Vasconcello-Castillo L . Assessment of activities of daily living in patients post COVID-19: a systematic review. PeerJ. (2021) 9:e11026. doi: 10.7717/peerj.11026, 33868804 PMC8034364

[36] Wan X Wang W Liu J Tong T. Estimating the sample mean and standard deviation from the sample size, median, range and/or interquartile range. BMC Med Res Methodol. (2014) 14:135. doi: 10.1186/1471-2288-14-135, 25524443 PMC4383202

[37] Chinn S. A simple method for converting an odds ratio to effect size for use in meta-analysis. Stat Med. (2000) 19:3127–31. doi: 10.1002/1097-0258(20001130)19:22<3127::AID-SIM784>3.0.CO;2-M, 11113947

[38] Faul F Erdfelder E Lang A-G Buchner A. G*power 3: a flexible statistical power analysis program for the social, behavioral, and biomedical sciences. Behav Res Methods. (2007) 39:175–91. doi: 10.3758/BF03193146, 17695343

[39] Higgins JP Thompson SG. Quantifying heterogeneity in a meta-analysis. Stat Med. (2002) 21:1539–58. doi: 10.1002/sim.1186, 12111919

[40] Higgins JP Thompson SG Deeks JJ Altman DG. Measuring inconsistency in meta-analyses. BMJ. (2003) 327:557–60. doi: 10.1136/bmj.327.7414.557, 12958120 PMC192859

[41] Darbandi M Hamzeh B Ayenepour A Rezaeian S Najafi F Shakiba E . Anti-inflammatory diet consumption reduced fatty liver indices. Sci Rep. (2021) 11:22601. doi: 10.1038/s41598-021-98685-3, 34799655 PMC8604894

[42] Rezazadegan M Shirani M Samadanian F Akbari M Shirani F. Association between dietary inflammatory index and phase angle in university employees: a cross-sectional study. Sci Rep. (2024) 14:21664. doi: 10.1038/s41598-024-71855-9, 39289398 PMC11408530

[43] Li L Shu X Yi Y Wang C Li J Ding Y . Dietary inflammatory impact on NAFLD development in obese vs. lean individuals: an analysis based on NHANES 2003-2018. Lipids Health Dis. (2024) 23:127. doi: 10.1186/s12944-024-02082-4, 38685122 PMC11619212

[44] Ruiz-Canela M Zazpe I Shivappa N Hébert JR Sánchez-Tainta A Corella D . Dietary inflammatory index and anthropometric measures of obesity in a population sample at high cardiovascular risk from the PREDIMED (PREvención con DIeta MEDiterránea) trial. Br J Nutr. (2015) 113:984–95. doi: 10.1017/s0007114514004401, 25720588 PMC4870040

[45] Mazidi M Shivappa N Wirth MD Hebert JR Mikhailidis DP Kengne AP . Dietary inflammatory index and cardiometabolic risk in US adults. Atherosclerosis. (2018) 276:23–7. doi: 10.1016/j.atherosclerosis.2018.02.020, 30015256

[46] Correa-Rodríguez M Rueda-Medina B González-Jiménez E Correa-Bautista JE Ramírez-Vélez R Schmidt-RioValle J. Dietary inflammatory index, bone health and body composition in a population of young adults: a cross-sectional study. Int J Food Sci Nutr. (2018) 69:1013–9. doi: 10.1080/09637486.2018.1446915, 29513154

[47] Toğuç H Öngün Yılmaz H Yaprak B. Exploring the link between dietary inflammatory index, inflammatory biomarkers, and sleep quality in adults with obesity: a pilot investigation. Int J Obes. (2025) 49:1037–42. doi: 10.1038/s41366-025-01728-2, 39885337 PMC12158772

[48] Corrêa CR da Costa BGG Silva KS Shivappa N Wirth MD Hébert JR . A higher energy-adjusted dietary inflammatory index is positively associated with total and visceral body fat in young male adults. J Hum Nutr Diet. (2022) 35:1136–50. doi: 10.1111/jhn.13012, 35377488

[49] Zhang Y Liu X Su Y Jiang Y Cai J Yang X . The relationship between dietary inflammatory index and metabolic syndrome and its components: a case study in Kashi urban, Xinjiang. Front Nutr. (2024) 11:1334506. doi: 10.3389/fnut.2024.1334506, 38487635 PMC10937582

[50] Camargo-Ramos CM Correa-Bautista JE Correa-Rodríguez M Ramírez-Vélez R. Dietary inflammatory index and cardiometabolic risk parameters in overweight and sedentary subjects. Int J Environ Res Public Health. (2017) 14:1104. doi: 10.3390/ijerph14101104, 28984835 PMC5664605

[51] Zhao Q Tan X Su Z Manzi HP Su L Tang Z . The relationship between the dietary inflammatory index (DII) and metabolic syndrome (MetS) in middle-aged and elderly individuals in the United States. Nutrients. (2023) 15:1857. doi: 10.3390/nu15081857, 37111075 PMC10146265

[52] Rahimlou M Ahmadi AR Cheraghian B Baghdadi G Ghalishourani SS Nozarian S . The association between dietary inflammatory index with some cardio-metabolic risk indices among the patients with type 2 diabetes from Hoveyzeh cohort study: a cross-sectional study. BMC Endocr Disord. (2024) 24:91. doi: 10.1186/s12902-024-01624-2, 38890603 PMC11186237

[53] Mokhtary N Mousavi SN Sotoudeh G Qorbani M Kalantar Z Koohdani F. Association between dietary inflammatory indices (DII, EDII) and obesity with consideration of insertion/deletion Apo B polymorphism in type 2 diabetic patients. Obes Med. (2020) 19:100241. doi: 10.1016/j.obmed.2020.100241, 38826717

[54] Kim HY Lee J Kim J. Association between dietary inflammatory index and metabolic syndrome in the general Korean population. Nutrients. (2018) 10:648. doi: 10.3390/nu10050648, 29883378 PMC5986527

[55] Shu L Zhao YY Shen YQ Zhang JY Li L. The dietary inflammatory index and metabolic health of population-based Chinese elderly. Asia Pac J Clin Nutr. (2022) 31:305–11. doi: 10.6133/apjcn.202206_31(2).0016, 35766566

[56] Wang YB Shivappa N Hébert JR Page AJ Gill TK Melaku YA. Association between dietary inflammatory index, dietary patterns, plant-based dietary index and the risk of obesity. Nutrients. (2021) 13:1536. doi: 10.3390/nu13051536, 34063221 PMC8147427

[57] Hodge AM Karim MN Hébert JR Shivappa N Milne RL de Courten B. Diet scores and prediction of general and abdominal obesity in the Melbourne collaborative cohort study. Public Health Nutr. (2021) 24:6157–68. doi: 10.1017/s1368980021001713, 33875030 PMC11148580

[58] Nouri-Majd S Salari-Moghaddam A Keshteli AH Esmaillzadeh A Adibi P. Dietary inflammatory potential in relation to general and abdominal obesity. Int J Clin Pract. (2022) 2022:5685249. doi: 10.1155/2022/5685249, 35685556 PMC9159184

[59] Schleh MW Caslin HL Garcia JN Mashayekhi M Srivastava G Bradley AB . Metaflammation in obesity and its therapeutic targeting. Sci Transl Med. (2023) 15:eadf9382. doi: 10.1126/scitranslmed.adf9382, 37992150 PMC10847980

[60] Tuomisto K Jousilahti P Havulinna AS Borodulin K Männistö S Salomaa V. Role of inflammation markers in the prediction of weight gain and development of obesity in adults - a prospective study. Metabol Open. (2019) 3:100016. doi: 10.1016/j.metop.2019.100016, 32812925 PMC7424817

[61] Barzilay JI Forsberg C Heckbert SR Cushman M Newman AB. The association of markers of inflammation with weight change in older adults: the cardiovascular health study. Int J Obes. (2006) 30:1362–7. doi: 10.1038/sj.ijo.0803306, 16534520

[62] Barbaresko J Koch M Schulze MB Nöthlings U. Dietary pattern analysis and biomarkers of low-grade inflammation: a systematic literature review. Nutr Rev. (2013) 71:511–27. doi: 10.1111/nure.12035, 23865797

[63] Holz T Thorand B Döring A Schneider A Meisinger C Koenig W. Markers of inflammation and weight change in middle-aged adults: results from the prospective MONICA/KORA S3/F3 study. Obesity. (2010) 18:2347–53. doi: 10.1038/oby.2010.73, 20360759

[64] Albracht-Schulte K Kalupahana NS Ramalingam L Wang S Rahman SM Robert-McComb J . Omega-3 fatty acids in obesity and metabolic syndrome: a mechanistic update. J Nutr Biochem. (2018) 58:1–16. doi: 10.1016/j.jnutbio.2018.02.012, 29621669 PMC7561009

[65] Cantero I Abete I Babio N Arós F Corella D Estruch R . Dietary inflammatory index and liver status in subjects with different adiposity levels within the PREDIMED trial. Clin Nutr. (2018) 37:1736–43. doi: 10.1016/j.clnu.2017.06.027, 28734553

[66] Xu Y Zhang M Wu T Dai S Xu J Zhou Z. The anti-obesity effect of green tea polysaccharides, polyphenols and caffeine in rats fed with a high-fat diet. Food Funct. (2015) 6:296–303. doi: 10.1039/c4fo00970c, 25431018

[67] Lozano CP Wilkens LR Shvetsov YB Maskarinec G Park S-Y Shepherd JA . Associations of the dietary inflammatory index with total adiposity and ectopic fat through the gut microbiota, LPS, and C-reactive protein in the multiethnic cohort–adiposity phenotype study. Am J Clin Nutr. (2022) 115:1344–56. doi: 10.1093/ajcn/nqab398, 34871345 PMC9071464

[68] Shafiee NH Razalli NH Shahril MR Muhammad Nawawi KN Mohd Mokhtar N Abd Rashid AA . Dietary inflammatory index, obesity, and the incidence of colorectal cancer: findings from a hospital-based case-control study in Malaysia. Nutrients. (2023) 15:982. doi: 10.3390/nu15040982, 36839339 PMC9965675

[69] Wu Y Li D Vermund SH. Advantages and limitations of the body mass index (BMI) to assess adult obesity. Int J Environ Res Public Health. (2024) 21:757. doi: 10.3390/ijerph21060757, 38929003 PMC11204233

[70] Shu Y Wu X Wang J Ma X Li H Xiang Y. Associations of dietary inflammatory index with prediabetes and insulin resistance. Front Endocrinol. (2022) 13:2022. doi: 10.3389/fendo.2022.820932, 35250879 PMC8892213

[71] Zhang X Guo Y Yao N Wang L Sun M Xu X . Association between dietary inflammatory index and metabolic syndrome: analysis of the NHANES 2005-2016. Front Nutr. (2022) 9:991907. doi: 10.3389/fnut.2022.991907, 36276824 PMC9582939

[72] Chen L-W Aubert AM Shivappa N Bernard JY Mensink-Bout SM Geraghty AA . Maternal dietary quality, inflammatory potential and childhood adiposity: an individual participant data pooled analysis of seven European cohorts in the ALPHABET consortium. BMC Med. (2021) 19:33. doi: 10.1186/s12916-021-01908-7, 33612114 PMC7898733

[73] Neufcourt L Assmann KE Fezeu LK Touvier M Graffouillère L Shivappa N . Prospective association between the dietary inflammatory index and metabolic syndrome: findings from the SU.VI.MAX study. Nutr Metab Cardiovasc Dis. (2015) 25:988–96. doi: 10.1016/j.numecd.2015.09.002, 26482566

[74] Howes EM Parker MK Misyak SA DiFeliceantonio AG Davy BM Brown LEC . The impact of weight bias and stigma on the 24 h dietary recall process in adults with overweight and obesity: a pilot study. Nutrients. (2024) 16:191. doi: 10.3390/nu16020191, 38257084 PMC10818297

[75] Whitton C Healy JD Dhaliwal SS Shoneye C Harray AJ Mullan BA . Demographic and psychosocial correlates of measurement error and reactivity bias in a 4-d image-based mobile food record among adults with overweight and obesity. Br J Nutr. (2023) 129:725–36. doi: 10.1017/S0007114522001532, 35587722 PMC9899562

[76] Kenđel Jovanović GM-SI Pavičić Žeželj S Šuša B Rahelić D Klobučar Majanović S. The efficacy of an energy-restricted anti-inflammatory diet for the management of obesity in younger adults. Nutrients. (2020) 12:3583. doi: 10.3390/nu12113583, 33266499 PMC7700374

[77] Salama AAAE Salem HA Abd El Fattah NK. Anti-inflammatory dietary combo in overweight and obese women with polycystic ovary syndrome. N Am J Med Sci. (2015) 7:310–6. doi: 10.4103/1947-2714.161246, 26258078 PMC4525389

[78] Lachat, C., Hawwash, D., Ocké, M.C., Berg, C., Forsum, E., Hörnell, A., Larsson, C.L., Sonestedt, E., Wirfält, E., Åkesson, A. and Kolsteren, P. Strengthening the reporting of observational studies in epidemiology-nutritional epidemiology (STROBE-nut): an extension of the STROBE statement. PLoS Med (2016) 13:e1002036. doi:10.1371/journal.pmed.1002036 27270749 PMC4896435

[79] Vermeulen SJ Park T Khoury CK Béné C. Changing diets and the transformation of the global food system. Ann N Y Acad Sci. (2020) 1478:3–17. doi: 10.1111/nyas.14446, 32713024 PMC7689688

[80] Dong W Man Q Zhang J Liu Z Gong W Zhao L . Geographic disparities of dietary inflammatory index and its association with hypertension in middle-aged and elders in China: results from a nationwide cross-sectional study. Front Nutr. (2024) 11:1355091. doi: 10.3389/fnut.2024.1355091, 38515520 PMC10955052

[81] Molag ML de Vries JH Ocké MC Dagnelie PC van den Brandt PA Jansen MC . Design characteristics of food frequency questionnaires in relation to their validity. Am J Epidemiol. (2007) 166:1468–78. doi: 10.1093/aje/kwm236, 17881382

[82] El Kinany K Garcia-Larsen V Khalis M Deoula MMS Benslimane A Ibrahim A . Adaptation and validation of a food frequency questionnaire (FFQ) to assess dietary intake in Moroccan adults. Nutr J. (2018) 17:61. doi: 10.1186/s12937-018-0368-4, 29895304 PMC5998554

