# Supplementary material for: High dietary inflammatory index is associated with an increased risk of overweight and obesity in adults: a meta-analysis of observational studies
Source: Front Nutr. 2026 Jun 30;13:1881176. doi: 10.3389/fnut.2026.1881176 (PMC13365322; doi:10.3389/fnut.2026.1881176)
Supplement: Supplementary file 1 [file Table_1.DOCX]

**Table S1.** Method of the database search strategy using PubMed, Embase, Web of Science, Science Direct and Cochrane

| **Database** | **Search terms and strategies** | **Number of studies searched** |
| --- | --- | --- |
| PubMed | (((((dietary inflammatory index[Title/Abstract]) OR (dietary inflammatory potential[Title/Abstract])) OR (anti-inflammatory diet[Title/Abstract])) OR (pro-inflammatory diet[Title/Abstract])))) AND ((((((((((("obesity"[Mesh]) OR (overweight[Title/Abstract])) OR (anthropometric measurements[Title/Abstract])) OR (body mass index[Title/Abstract])) OR (excess weight[Title/Abstract])) OR (adiposity[Title/Abstract])) OR (abdominal obesity[Title/Abstract])) OR (body fat[Title/Abstract])) OR (obesity management [Title/Abstract])) OR (BMI [Title/Abstract]))) AND ("adult"[Mesh]) | 301 |
| Embase | (adult:ti,ab,kw) AND ('dietary inflammatory index':ti,ab,kw OR 'dietary inflammatory potential':ti,ab,kw OR 'anti-inflammatory diet':ti,ab,kw OR 'pro-inflammatory diet':ti,ab,kw) AND (('obesity'/exp) OR ('adipose tissue hyperplasia':ti,ab,kw OR adipositas:ti,ab,kw OR adiposity:ti,ab,kw OR 'alimentary obesity':ti,ab,kw OR 'body weight, excess':ti,ab,kw OR corpulency:ti,ab,kw OR 'fat overload syndrome':ti,ab,kw OR 'nutritional obesity':ti,ab,kw OR obesitas:ti,ab,kw OR overweight:ti,ab,kw OR obesity:ti,ab,kw OR 'central obesity':ti,ab,kw OR 'abdominal obesity':ti,ab,kw)) | 32 |
| Web of Science | TS=(adult) AND (((TS=(dietary inflammatory index)) OR TS=(dietary inflammatory potential)) OR TS=(anti-inflammatory diet)) OR (TS=(pro-inflammatory diet) AND ((((((((((TS=(obesity)) OR TS=(overweight)) OR TS=(obesity, abdominal)) OR TS=(central obesity)) OR TS=(abdominal obesity)) OR TS=(anthropometric measurements)) OR TS=(excess weight)) OR TS=(adiposity)) OR TS=(body mass index)) OR TS=(body fat)) OR TS=(obesity management)) | 1,011 |
| Science Direct | adult AND ("dietary inflammatory index" OR "dietary inflammatory potential" OR "anti-inflammatory diet" OR "pro-inflammatory diet") AND ("obesity" OR "overweight" OR "central obesity" OR " body mass index " ) | 44 |
| Cochrane | (MeSH descriptor: [adult] explode all trees) AND ((dietary inflammatory index):ti,ab,kw OR (dietary inflammatory potential):ti,ab,kw OR (anti-inflammatory diet):ti,ab,kw OR (pro-inflammatory diet):ti,ab,kw) AND ((MeSH descriptor: [obesity] explode all trees) OR ((overweight):ti,ab,kw OR (obesity, abdominal):ti,ab,kw OR (central obesity):ti,ab,kw OR (abdominal obesity):ti,ab,kw OR (anthropometric measurements):ti,ab,kw) OR ((excess weight):ti,ab,kw OR (adiposity):ti,ab,kw OR (body mass index):ti,ab,kw OR (body fat):ti,ab,kw OR (obesity management):ti,ab,kw) OR ((excess weight):ti,ab,kw OR (adiposity):ti,ab,kw OR (body mass index):ti,ab,kw OR (body fat):ti,ab,kw OR (obesity management):ti,ab,kw)) | 573 |

**Table S2.** Quality of included studies was assessed using the NIH Quality Assessment Tool for Observational Cohort and Cross-Sectional Studies

| **First author (year)** | **1** | **2** | **3** | **4** | **5** | **6** | **7** | **8** | **9** | **10** | **11** | **12** | **13** | **14** | **Total Score** | **Quality Rating** |
| --- | --- | --- | --- | --- | --- | --- | --- | --- | --- | --- | --- | --- | --- | --- | --- | --- |
| Darbandi (2021) | Y | Y | Y | Y | N | N | N | Y | Y | N | Y | NR | NA | Y | 8/13（61.5%） | Fair |
| Rezazadegan (2024) | Y | Y | Y | Y | Y | N | N | Y | Y | N | Y | NR | NA | Y | 9/13（69.2%） | Fair |
| Karimbeiki (2021) | Y | Y | Y | Y | N | N | N | N | Y | N | Y | NR | NA | Y | 7/13（53.8%） | Fair |
| Shi (2023) | Y | Y | Y | Y | N | N | N | Y | Y | N | Y | NR | NA | Y | 8/13（61.5%） | Fair |
| San (2018) | Y | Y | Y | Y | N | N | N | Y | Y | N | Y | NR | NA | Y | 7/13（53.8%） | Fair |
| Li (2024) | Y | Y | N | Y | N | N | N | Y | Y | N | Y | NR | NA | Y | 7/13（53.8%） | Fair |
| Ruiz-Canela (2015) | Y | Y | Y | Y | N | N | N | Y | Y | N | Y | NR | NA | Y | 8/13（61.5%） | Fair |
| Mazidi (2018) | Y | Y | Y | Y | N | N | N | Y | Y | N | Y | NR | NA | Y | 8/13（61.5%） | Fair |
| Muhammad (2019) | Y | Y | Y | Y | N | N | N | Y | Y | N | Y | NR | NA | Y | 8/13（61.5%） | Fair |
| Correa-Rodríguez (2018) | Y | Y | Y | Y | N | N | N | Y | Y | N | Y | NR | NA | Y | 8/13（61.5%） | Fair |
| Nouri-Majd (2022) | Y | Y | CD | Y | N | N | N | Y | Y | N | N | NR | NA | Y | 6/13（46.2%） | Poor |
| Togug (2025) | Y | Y | Y | Y | Y | N | N | Y | Y | N | N | NR | NA | N | 7/13（53.8%） | Fair |
| Corrêa (2022) | Y | Y | Y | Y | Y | N | N | Y | Y | N | Y | NR | NA | Y | 9/13（69.2%） | Fair |
| Zhang (2024) | Y | Y | Y | Y | N | N | N | Y | Y | N | Y | NR | NA | Y | 8/13（61.5%） | Fair |
| Zhao (2023) | Y | Y | Y | Y | N | N | N | Y | Y | N | Y | NR | NA | Y | 8/13（61.5%） | Fair |
| Rahimlou (2024) | Y | Y | Y | Y | N | N | N | Y | Y | N | Y | NR | NA | Y | 8/13（61.5%） | Fair |
| Mokhtary (2020) | Y | Y | Y | Y | N | N | N | Y | Y | N | Y | NR | NA | Y | 8/13（61.5%） | Fair |
| Camargo-Ramos (2017) | Y | Y | Y | Y | N | N | N | Y | Y | N | Y | NR | NA | Y | 8/13（61.5%） | Fair |
| Kim (2018) | Y | Y | Y | Y | N | N | N | Y | Y | N | Y | NR | NA | Y | 8/13（61.5%） | Fair |
| Shu (2022) | Y | Y | Y | Y | N | N | N | Y | Y | N | Y | NR | NA | Y | 8/13（61.5%） | Fair |
| Wang (2021) | Y | Y | Y | Y | Y | N | N | Y | Y | N | Y | NR | N | Y | 11/14（78.6%） | Good |
| Hodge (2021) | Y | Y | Y | Y | N | Y | Y | Y | Y | N | Y | NR | N | Y | 10/14（71.4%） | Fair |
| Ramallal (2017) | Y | Y | Y | Y | N | Y | Y | Y | Y | Y | N | NR | N | Y | 10/14（71.4%） | Fair |

**Additional Comments:** The study by Nouri-Majd S (2022) was rated as poor because the outcome data were self-reported by the participants, and the overall quality score was below 50%.

**Quality of included studies was assessed using the National Institutes of Health (NIH) Quality Assessment tool for Observational Cohort and Cross-Sectional Studies** (<https://www.nhlbi.nih.gov/health-pro/guidelines/in-develop/cardiovascular-risk-reduction/tools/cohort>).

1. Was the research question or objective in this paper clearly stated?

2. Was the study population clearly specified and defined?

3. Was the participation rate of eligible persons at least 50%?

4. Were all the subjects selected or recruited from the same or similar populations (including the same time period)? Were inclusion and exclusion criteria for being in the study prespecified and applied uniformly to all participants?

5. Was a sample size justification, power description, or variance and effect estimates provided?

6. For the analyses in this paper, were the exposure(s) of interest measured prior to the outcome(s) being measured?

7. Was the timeframe sufficient so that one could reasonably expect to see an association between exposure and outcome if it existed?

8. For exposures that can vary in amount or level, did the study examine different levels of the exposure as related to the outcome (e.g., categories of exposure, or exposure measured as continuous variable)?

9. Were the exposure measures (independent variables) clearly defined, valid, reliable, and implemented consistently across all study participants?

10. Was the exposure(s) assessed more than once over time?

11. Were the outcome measures (dependent variables) clearly defined, valid, reliable, and implemented consistently across all study participants?

12. Were the outcome assessors blinded to the exposure status of participants?

13. Was loss to follow-up after baseline 20% or less?

14. Were key potential confounding variables measured and adjusted statistically for their impact on the relationship between exposure(s) and outcome(s)?

**Total Score**, number of yes; **NA**, not applicable; **NR**, not reported; **CD**, cannot determine; **Y**: Yes, present; **N**: No.

**Quality Rating:** Poor <50%, Fair ≥50% and <75%, Good ≥75%.

**Table S3.** Subgroup analysis according to the study characteristics

| **Subgroup** | **Number of studies** | **Effect size value (95% CI)** ^a^ | ***I*² value** | ***P value***  **(Q test)** ^b^ | ***P* value (Heterogeneity between groups)** ^c^ |
| --- | --- | --- | --- | --- | --- |
| **Total** | 19 | Cohen’s d = 0.17 (95% CI: 0.09, 0.25) | 85.70% | < 0.001 |  |
| **WHO regions** | | | | | 0.08 |
| EMR | 6 | Cohen’s d = 0.22 (95% CI: 0.15, 0.29) | 23.00% | 0.26 |  |
| AMR | 6 | Cohen’s d = 0.25 (95% CI: 0.20, 0.30) | 15.60% | 0.31 |  |
| EUR | 2 | Cohen’s d = 0.09 (95% CI: –0.10, 0.29) | 65.90% | 0.09 |  |
| SEAR | 2 | Cohen’s d = 0.09 (95% CI: –0.08, 0.27) | 0.00% | 0.42 |  |
| WPR | 3 | Cohen’s d = 0.02 (95% CI: –0.20, 0.23) | 94.80% | < 0.001 |  |
| **Population health status** | | | | | 0.52 |
| Unhealthy | 11 | Cohen’s d = 0.15 (95% CI: 0.06, 0.24) | 90.30% | < 0.001 |  |
| Healthy | 8 | Cohen’s d = 0.21 (95% CI: 0.07, 0.34) | 49.90% | 0.052 |  |
| **Dietary assessment tool** | | | | | 0.28 |
| FFQ | 9 | Cohen’s d = 0.18 (95% CI: 0.14, 0.22) | 19.60% | 0.27 |  |
| 24-HDR | 9 | Cohen’s d = 0.20 (95% CI: 0.04, 0.35) | 92.20% | < .001 |  |
| FFQ + 24-HDR | 1 | Cohen’s d = 0.03 (95% CI: –0.16, 0.22) | |  |  |
| **DII component parameters** | | | | | 0.44 |
| ≥30 | 9 | Cohen’s d = 0.21 (95% CI: 0.16, 0.26) | 44.40% | 0.06 |  |
| <30 | 10 | Cohen’s d = 0.14 (95% CI: 0.00, 0.29) | 89.70% | < 0.001 |  |
| **DII group category** | | | | | 0.85 |
| Quartile | 10 | Cohen’s d = 0.15 (95% CI: 0.05, 0.26) | 91.80% | < 0.001 |  |
| Tertile | 6 | Cohen’s d = 0.20 (95% CI: 0.06, 0.34) | 48.90% | 0.08 |  |
| Other | 3 | Cohen’s d = 0.17 (95% CI: 0.11, 0.24) | 0.00% | 0.80 |  |
| **Effect size category** | | | | | 0.34 |
| OR | 6 | Cohen’s d = 0.22 (95% CI: 0.14, 0.29) | 42.60% | 0.12 |  |
| SMD | 13 | Cohen’s d = 0.16 (95% CI: 0.06, 0.25) | 88.50% | < 0.001 |  |
| **Publication year** | | | | | 0.040 |
| >2020 | 11 | Cohen’s d = 0.23 (95% CI: 0.18, 0.28) | 46.00% | 0.047 |  |
| ≤2020 | 8 | Cohen’s d = 0.08 (95% CI: –0.05, 0.21) | 88.70% | < 0.001 |  |
| **Female proportion (%) ^d^** | | | | | 0.27 |
| ≤50% | 5 | Cohen’s d = 0.22 (95% CI: 0.14, 0.26) | 27.70% | 0.24 |  |
| >50% | 13 | Cohen’s d = 0.15 (95% CI: 0.05, 0.25) | 88.80% | < 0.001 |  |
| **DII/E-DII** | | | | | 0.98 |
| DII | 16 | Cohen’s d = 0.17 (95% CI: 0.08, 0.25) | 87.60% | < 0.001 |  |
| E-DII | 3 | Cohen’s d = 0.17 (95% CI: 0.02, 0.32) | 47.30% | 0.15 |  |

^a^ Values were obtained from random-effects model.

^b^ *P* for Q test obtained from random-effects model.

^c^ *P* for heterogeneity, between subgroups obtained from random-effects model.

^d^ One study did not report the proportion of female or male participants.

Abbreviations: EMR: Eastern Mediterranean Region; AMR: Region of the Americas; EUR: European Region; SEAR: South-East Asia Region; WPR: Western Pacific Region; OR: odds ratio; SMD: standardized mean difference (Cohen’s d); CIs: confidence intervals; FFQ: food frequency questionnaire; 24-HDR: 24-hour dietary recall.

**Table S4.** Meta-regression analysis by study characteristics

| **Covariate** | **β** | **95% CI** | ***P*-value** |
| --- | --- | --- | --- |
| Effect size category | –0.057 | –0.213, 0.099 | .45 |
| WHO regions | –0.060 | –0.098, –0.022 | .004 |
| Population health status | 0.529 | –0.113, 0.219 | .51 |
| Dietary assessment tool | –0.034 | –0.159, 0.090 | .57 |
| DII component parameters | 0.006 | –0.001, 0.013 | .11 |
| DII group category | –0.008 | –0.105, 0.090 | .87 |
| Publication year | 0.160 | 0.034, 0.287 | .016 |
| Sample size | –6.26E-07 | –0.0000158, 0.0000145 | .93 |
| Mean age | 0.002 | –0.005, 0.010 | .52 |
| Female proportion (%) | –0.393 | –1.029, 0.243 | .21 |

Abbreviations: β: regression coefficient.


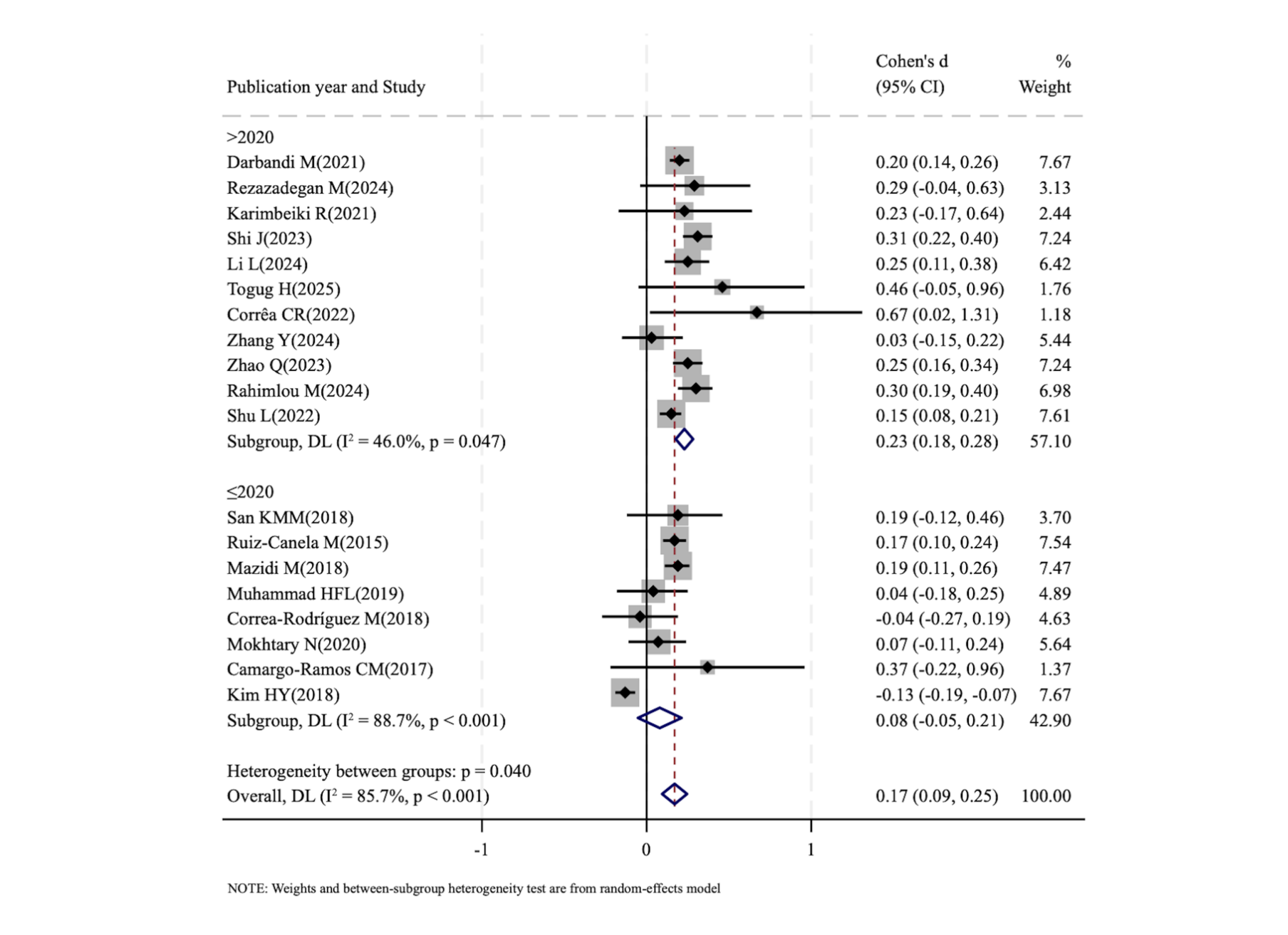


**Figure S1.** Forest plot of the association between the highest dietary inflammatory index and the risk of overweight/obesity, stratified by publication year.


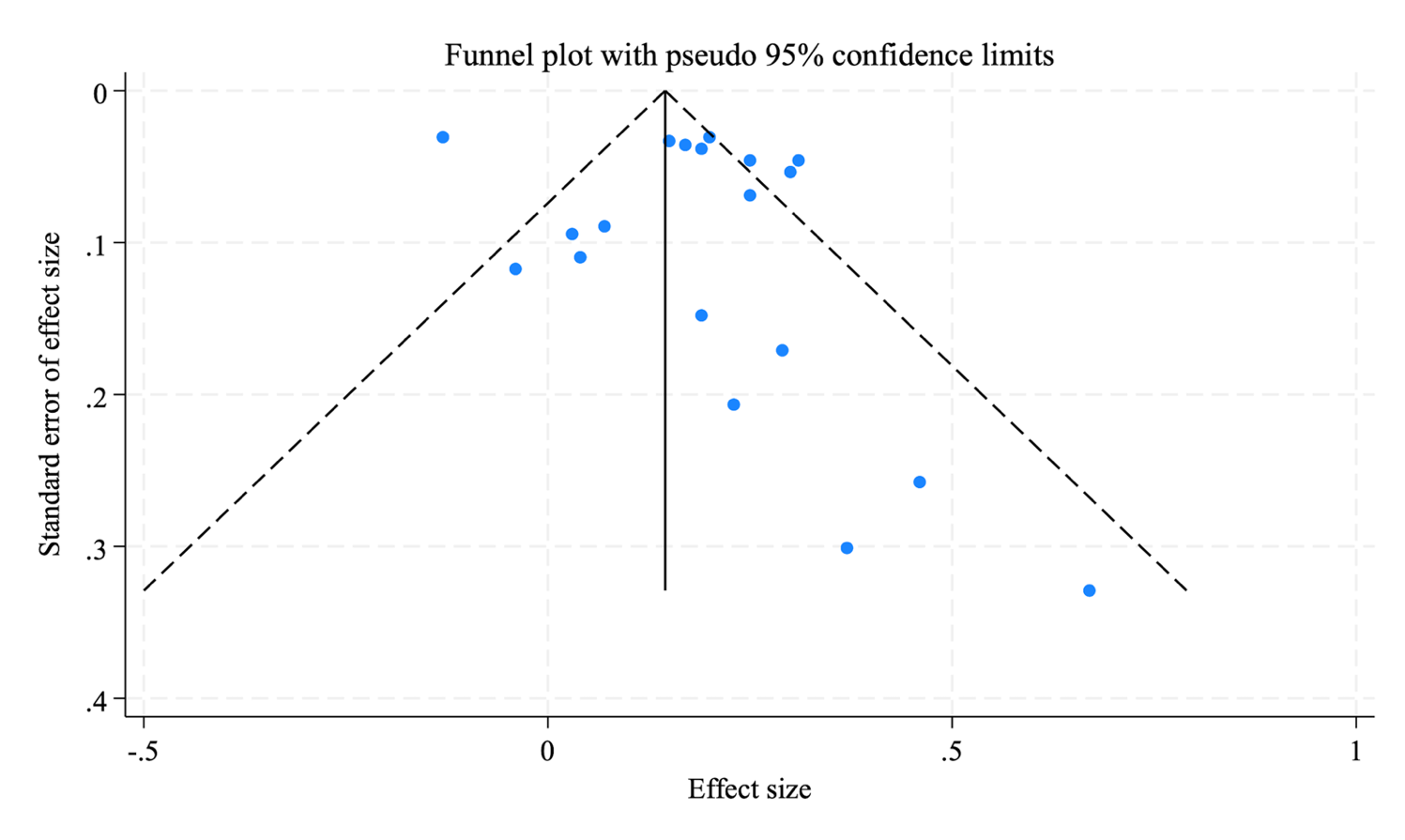


**Figure S2.** Results of funnel plot for the association between the dietary inflammatory index and the risk of overweight/obesity.
